# Rapid Dilatation of the Female Urethra in the Diagnosis and Treatment of Chronic Cystitis

**Published:** 1878-07

**Authors:** Wm. H. Byford


### RAPID DILATATION OF THE FEMALE URETHRA IN THE DIAGNOSIS AND TREATMENT OF CHRONIC CYSTITIS.

By Wm. H. Byford, M. D.

M. C., a German by birth, was 26 years old when I first saw her. She was the mother of two children, the first two years old, the second five months. She was short, stout and, up to the time of her first confinement, had enjoyed uninterrupted health. The confinement, however, was severe and somewhat protracted. She was attended by a midwife, and all that I have been able to ascertain of her history, pertinent to the present report, is that she suffered for several months during lactation from irritability of the bladder. Apparently, however, she had entirely recovered from this before her last pregnancy.

During the last two months of her second gestation she suffered from dysuria, which, as labor approached, was attended with marked symptoms of inflammation of the bladder, such as inability to retain the urine more than half an hour or an hour at a time, a burning pain and tenderness in the pubic region, with a sense of weight and tenesmus.

At the time I first saw her, just five months after the second child was born, she was standing with her head and upper part of the body bent forward and her feet widely separated, a position she maintained as much as possible, on account of the pain she experienced while sitting or lying. Every ten or fifteen minutes she was seized with great vesical tenesmus, which was attended with the forcible expulsion of a small quantity of urine. This evacuation of the bladder, although the urine was so small in quantity, would bring some relief for another ten or fifteen minutes, at which time occurred a similar paroxysm of pain and urinary discharge. In this way she passed the time until she became so exhausted with fatigue, suffering and wakefulness that she would be obliged to assume the recumbent position, and doze between her painful paroxysms. When she thus attempted to sleep she would lie but a few moments, as the pressure of a small amount of urine would arouse her—to secure its expulsion—by an uncontrollable tenesmus.

Several months of such agony imprinted upon her face marks of great suffering, and induced a general failure of mental and physical power. Her pulse was small, and about a hundred to the minute; skin cool and damp; tongue coated and appetite poor. The urine was retained so short a time, and expelled in such small quantities, that I could not procure any for examination. The external genitals were excoriated and covered with mucus, and large urinary deposits covered the labia and perineum. The labia were tumid and their inner surfaces so raw that they frequently bled. The parts were so tender that I found it impossible to make any examination without subjecting her to the influence of an anæsthetic.

When she was completely under the influence of ether, a large steel sound was introduced into the bladder and swept around in every direction without encountering anything solid therein. The forefinger of the right hand was then introduced through the urethra into the bladder, which was found to be so contracted that every part of its cavity could be easily explored. The walls were very thick and rigid, and the mucous membrane studded with pendulous granulations or projections, easily perceived by the touch. These were forcibly detached by the finger until, as far as possible, all were thus disposed of. Two or three ounces of blood were lost during this operation. The distension of the urethra was accomplished without rupturing any of the muscular fibers surrounding it. For fear of a too rapid contraction of the sphincters, the middle finger was introduced to still further dilate the urethra. I entertained no fear that this great distension of the urethra would result in the patient's inability to retain her urine. Indeed, I apprehended that it might be necessary before the cure was completed to repeat the dilatation.

After the operation was finished, Nov 15th, 2 p.m., the patient promptly and thoroughly recovered from the effects of the ether, and at 3 p. m. was comparatively comfortable. One drachm of acetate of potash every two hours was prescribed. An injection into the bladder was ordered of a quart of water at 112° Fahrenheit twice daily; and a tepid sitz bath for half an hour twice a day also. Nov. 16th; The injection last night caused slight pain, which very soon passed off, and was followed by a sense of comfort. The urine flowed freely through the dilated urethra, and the patient slept quite well a good portion of the night.

Nov. 17; The patient rested well, no pain, had free secretion and discharge of urine. Treatment to be continued. Nov. 18; Still improving, and expresses herself as greatly relieved from the suffering she experienced before the operation. Nov. 20; Patient still improving in every respect. The excoriations of the external genitals are disappearing; she now retains her urine two hours, and passes it without discomfort. Nov. 24; The treatment has not been changed and the patient continues to improve. She retains her urine for five or six hours without inconvenience. The catheter now passes three inches into the bladder, and the injection is a source of great comfort. The urine is clear and but little mucus settles at the bottom of the vessel. The potash is omitted to-day, and the patient directed to take one teaspoonful of the syrup of the tincture of iron after meals, three times a day. This syrup contains fifteen minims of the tincture to the teaspoonful. The sitz baths and injections are to be continued.

This treatment was pursued until Dec. 6th, when the patient left the hospital at her own request, believing that the treatment was no longer necessary.

Two years ago there was, in the Mercy Hospital, a case of severe chronic cystitis in a little girl ten years of age, in which it was necessary to dilate the urethra with the little finger four times. So far from this practice having been attended with any bad results, the little patient was discharged at the end of four months entirely cured, her ability to retain her urine being perfect. Up to this time the disease has not returned.

Three years ago, I saw an interesting trial of this operation in a very unfavorable case, in consultation with Prof. D. T. Nelson. The patient was an Irish woman, totally broken down in health from a complication of difficulties connected with chronic and very distressing cystitis. She was the mother of several children, had had difficult labors, and very poor attention ; and had been subject to symptoms referable to uterine and vesical disease for several years. She was, at the time when I saw her first, afflicted with fibrinous deposit in the omentum, which strongly resembled a tumor, the result of a somewhat acute attack of general peritonitis some months before. The spleen was enlarged, and from the great functional disturbance of the heart it was not altogether certain that that organ might not be affected with organic disease. She was greatly reduced in strength, and anasarcous to a moderate degree. She was constipated, had a poor appetite, and altogether justified the assertion I have just made, that she was totally broken down in health. The most distressing of her symptoms, however, she referred to her bladder. The dysuria was so severe and continuous, that she had no rest except under the influence of opiates. Large scales of phosphatic deposits, with pus, mucus and sometimes blood, were passed from the bladder with the urine in most agonizing paroxysms. In addition to the judicious treatment which was being pursued by Dr. N., I advised him to pass his finger into the bladder as often as necessary to keep the urethra sufficiently patent to permit of a free discharge of the contents of the bladder, and if necessary wash the organ out with warm water.

As soon as the dilatation was performed the patient began to improve, and the improvement steadily progressed. She is now in the enjoyment of a very fair condition of health. The disease of the bladder, however, was so serious as to require repeated dilatation for about a year from its commencement. The patient soon comprehended and appreciated the great relief afforded by introducing the finger through the urethra, and for a long time before she entirely recovered, when the irritation became very annoying, she would perform the operation herself.

The patient should be prepared for the operation of dilatation of the urethra, by thoroughly emptying the alimentary canal, by a liberal use of the warm bath; scrupulous cleanliness of the external genitals, and large injections of warm water into the bladder, by means of a double catheter twice a day for at least forty-eight hours.

We should always be careful before resorting to forcible dilatation of the urethra, to examine circumspectly the pelvic cavity and lower portion of the abdomen, in order to assure ourselves that there are none of the inflammations present which so often complicate vesical inflammation in the female pelvis, such as chronic or acute cellulitis, local peritonitis or a considerable amount of metritis.

Any of these complications would render the operation hazardous and would contra-indicate it.

I have been made acquainted with one instance, in which dilatation was performed, where peritonitis supervened in less than twenty-four hours, and, by becoming general, led to a fatal result. When none of these local complications are present, we may generally expect a favorable result. After dilatation, care must always be taken to exclude any of the ordinary causes of excessive reaction. This may be done by keeping the patient strictly quiet for three or four days, surrounding her with the best hygienic conditions, and relieving pain, should any occur, by administering full doses of opium. The bowels ought to be kept from moving for three or four days. At the end of this time gentle evacuations may be secured by repeated small doses of sulphate or citrate of magnesia. The diet for two or three days should be easy of digestion and nourishing.

The operation is very simple and easily accomplished, usually requiring not more than ten minutes for its performance. The one thing to be avoided is too much violence. Generally it is best to begin by pressing the end of the little finger against the meatus with gentleness, firmness and steadiness. In a short time, the muscular fibres are felt beginning to relax, and very soon they will permit the introduction of the finger. Having held the little finger in this position for a few minutes, it should be withdrawn, and the index finger introduced with the same precaution, giving the muscular fibres time to relax and permit the finger to enter, instead of urging it forward so rapidly as to rupture them.

